# Words in the bilingual brain: an fNIRS brain imaging investigation of lexical processing in sign-speech bimodal bilinguals

**DOI:** 10.3389/fnhum.2014.00606

**Published:** 2014-08-21

**Authors:** Ioulia Kovelman, Mark H. Shalinsky, Melody S. Berens, Laura-Ann Petitto

**Affiliations:** ^1^Department of Psychology, Psychology and Center for Human Growth and Development, University of MichiganAnn Arbor, MI, USA; ^2^U.S. Department of DefenseWashington, DC, USA; ^3^Visual Language and Visual Learning (VL2), Science of Learning Center, Gallaudet University, National Science FoundationWashington, DC, USA

**Keywords:** language, bilingualism, brain plasticity, functional near infrared spectroscopy brain imaging, fNIRS, “neural signature hypothesis”

## Abstract

Early bilingual exposure, especially exposure to two languages in different modalities such as speech and sign, can profoundly affect an individual's language, culture, and cognition. Here we explore the hypothesis that *bimodal* dual language exposure can also affect the brain's organization for language. These changes occur across brain regions universally important for language and parietal regions especially critical for sign language (Newman et al., 2002). We investigated three groups of participants (*N* = 29) that completed a word repetition task in American Sign Language (ASL) during fNIRS brain imaging. Those groups were (1) hearing ASL-English bimodal bilinguals (*n* = 5), (2) deaf ASL signers (*n* = 7), and (3) English monolinguals naïve to sign language (*n* = 17). The key finding of the present study is that bimodal bilinguals showed reduced activation in left parietal regions relative to deaf ASL signers when asked to use only ASL. In contrast, this group of bimodal signers showed greater activation in left temporo-parietal regions relative to English monolinguals when asked to switch between their two languages (Kovelman et al., 2009). Converging evidence now suggest that bimodal bilingual experience changes the brain bases of language, including the left temporo-parietal regions known to be critical for sign language processing (Emmorey et al., 2007). The results provide insight into the resilience and constraints of neural plasticity for language and bilingualism.

## Introduction

Studying bilinguals for whom sign language is one of their two languages provides a better way of understanding both the *resilience* and *plasticity* of human language and the underlying brain regions that support it. For example, left hemisphere inferior frontal and temporal regions demonstrate *resilience* in their support of language functions despite variations in language modality (signed or spoken) or the number of languages used (monolingual or bilingual; Petitto et al., 2000; MacSweeney et al., 2002a,b; Corina et al., 2003; Penhune et al., 2003; Kovelman et al., 2009; Emmorey et al., 2011). In contrast, the parietal brain regions, classically associated with visuo-spatial processing, show evidence of *plasticity* and become specialized for processing sign language structure (Neville et al., 1997; Newman et al., 2002; Emmorey et al., 2005). However, our understanding of how the brain accommodates sign language and bilingualism is incomplete without a better appreciation of *bimodal* bilinguals, bilinguals who can both hear a spoken language and learn a sign language, from early life (Petitto et al., 2000; Emmorey and McCullough, 2009). Here we explore the resilience and plasticity of the brain's organization for human language as an *outcome* of early bilingual language experience to a sign and a spoken language.

### Sign languages

*Sign languages* are visuo-spatial languages. Researchers have been investigating how early exposure to a sign vs. a spoken language may impact the brain. Bilateral parietal regions, known to be important for analyzing body motion and visuo-spatial orientation in hearing monolinguals, appear to become selectively engaged for sign language morpho-syntax (Corina et al., 1999), lexical retrieval (Emmorey et al., 2011), and word production (Emmorey et al., 2007). Importantly, there is also evidence of neuro-developmental plasticity, only adults who received *early* exposure to a sign language show right parietal recruitment during signing (Neville et al., 1997; Newman et al., 2002). Hence, researchers have suggested that learning a sign language across both deaf and hearing individuals may change the functionality of the parietal regions supporting sign language processing (Neville et al., 1997; Corina et al., 1999; Newman et al., 2002). Yet, it also remains possible that there are differences in functional language organization between deaf and bimodal signers in these resilient regions for language. These resilient areas include areas around classic Broca's and Wernicke's regions, as well as the plastic/parietal regions that support sign language use.

### Bilingualism

Neuroimaging research comparing bilingual and monolingual speakers of different languages generally support the idea that a “bilingual is not two monolinguals in one person,” (Grosjean, 1989; e.g., Abutalebi et al., 2001; Kovelman et al., 2009; Bialystok et al., 2012; Jasinska and Petitto, 2013; Klein et al., 2014). A key notable difference between bilinguals and monolinguals is the increase in greater gray matter density in left parietal regions in unimodal spoken language bilinguals relative to monolinguals (Mechelli et al., 2004). That study found that high proficiency bilinguals who learned their two languages before the age of 5 had the greatest increase in gray matter density relative to low proficiency and later exposed bilinguals, as well as monolinguals (Mechelli et al., 2004). Several studies have shown that unimodal spoken language bilinguals also typically show greater signal intensity in left inferior frontal regions during lexico-semantic (Kovelman et al., 2008c), syntactic (Jasinska and Petitto, 2013), and language switching tasks (Abutalebi et al., 2008; Kovelman et al., 2008c), relative to monolinguals. Yet, it remains generally unknown if these differences predominantly stem from frequent competition between bilinguals' two languages (Abutalebi et al., 2008) or the overall increase in language capacity to accommodate for two linguistic systems (Kovelman et al., 2008a). Research into bimodal bilinguals who potentially experience reduced competition between their two languages in non-overlapping auditory and visual modalities may shed new light on the nature of the brain's accommodation for two languages.

### Bimodal bilingualism

There are many critical similarities between bimodal and unimodal spoken language bilinguals. Specifically, young learners of a sign and a spoken language reach their first word and other linguistic milestones at the same time as unimodal bilinguals (Petitto et al., 2001a; Petitto and Kovelman, 2003; Petitto, 2005). Young bimodal and unimodal bilinguals also show similar trajectories of vocabulary growth across their two languages in the first 3 years of life (Holowka et al., 2002). Regarding language switching, bimodal bilinguals can use both languages at the same time while unimodal bilinguals can only use their two languages in alternation (because both languages cannot be expressed simultaneously from the mouth). On the other hand, it has been found that young bimodal bilinguals show unimodal-like patterns of language switching in response to their family's switching habits, interlocutor's language, and the morpho-syntactic structures of their languages (Petitto et al., 2001a; Petitto and Kovelman, 2003; Paradis and Nicoladis, 2007). Hence, one can hypothesize that all types of bilingualism, bimodal and unimodal, have a similar impact on language organization in the brain.

Nevertheless, it is possible that bimodal bilingualism makes a lesser impact on the bilingual brain relative to unimodal bilingualism. For instance, theories of bilingual cognition have put forth a hypothesis and supporting evidence that the necessity to switch between two languages from early in life can enhance a bilingual child's and adult's attentional capabilities (Bialystok, 2001, 2008; Bialystok et al., 2012; Garbin et al., 2010). However, this effect has not been found in young bimodal bilinguals (Emmorey et al., 2008), possibly because a sign and a spoken language compete less with each other over the sensorimotor mechanisms of language production and comprehension (Bialystok, 2001; Emmorey et al., 2008).

Another typical point of competition between two languages is lexico-semantic encoding of homophones, or words that sound the same across languages but can often mean different things. Specifically, *plain* and *plane* are English homophones that sound the same but have different meanings, the same phenomenon is possible across bilinguals' two different languages (Finkbeiner et al., 2006; Blumenfeld and Marian, 2013). Such type of language competition is not possible for bimodal bilinguals. Given the evidence that there is reduced levels of competition between sign and spoken languages relative to two spoken languages, could it then be possible that bimodal bilingualism does not change the brain beyond the specific accommodations for the person's sign and spoken languages?

What impact might dual language experience and language switching have for a bilingual brain? Neuroimaging studies of bilingual language switching have found greater activation in bilateral prefrontal and left temporo-parietal regions during language switching tasks in unimodal bilinguals as compared to using only one language at a time (Hernandez et al., 2001; Abutalebi et al., 2008; Kovelman et al., 2008c; Garbin et al., 2010). The only study that has investigated language switching in bimodal bilinguals found that this group only showed greater activation in left *temporo-parietal* regions during the switching condition as compared to non-switching and as compared to English monolinguals (Kovelman et al., 2009). In that study, we hypothesized that increased activation in temporo-parietal regions in bimodal bilinguals stemmed from the competition between bimodal bilinguals' lexico-semantic representations in the two languages (Kovelman et al., 2009). Nevertheless, it remains possible that increased activation in left the temporo-parietal region was due to the engagement of sign-specific language processes in the parietal regions (Newman et al., 2002), rather than the switching demands. The limitation of the previous study was that it only compared bimodal bilinguals to spoken language monolinguals. Hence, to fully answer the question on how the brain accommodates to bimodal bilingualism it is critical to also compare bimodal bilinguals to Deaf signers.

### Innovation of the present work

Most of the previous research examined the impact of early sign language exposure as compared to late sign language exposure or spoken language exposure (e.g., Neville et al., 1997; Newman et al., 2002). Here we explore the impact of early *bimodal bilingual* exposure to a sign and to a spoken language in *hearing* adults as compared to early ASL sign language exposure in deaf adults. The goal of the present study is to broaden our understanding of the resilience and plasticity of the brain's organization for language in light of both bilingual and bimodal exposure to two languages in different modalities.

### Hypothesis 1

There is limited sensory-motor and lexical competition between sign and spoken languages because they do not compete for the same sensory-motor apparatus and do not use similar phonetic features to encode words. Thus, the bimodal bilingual brain requires little additional neural adjustments for language processing beyond the plasticity necessary for accommodating the given spoken and sign languages. Therefore, one can predict *similarities* across ASL-English bilingual signers and unimodal deaf ASL signers.

### Hypothesis 2

Early bilingual exposure will lead to evidence of the brain's neural accommodation for processing two language systems, both in the brain's resilient regions that are universal for language processing (across sign and speech, including left frontal and temporal regions) and the plastic regions specific to sign language processing (including the parietal regions). Here, the neural conditions that give rise to being *bilingual* (the neural demands/impact of processing two languages) are governing the extent to which bilinguals will recruit brain regions that demonstrate universal resilience and language-specific plasticity. Thus, we can predict *differences* across bilingual ASL-English signers and functionally monolingual deaf ASL signers during the unimodal use of sign language.

The present study addressed these two hypotheses by testing three groups of participants: (1) native hearing ASL-English proficient bimodal bilinguals from birth, (2) native deaf signers of American Sign Language (ASL) who learned English as a second language but who are functionally monolingual deaf ASL signers, and (3) hearing English monolinguals naïve to sign. The participants completed a lexical/word repetition task in sign language which engages both language comprehension and language production mechanisms. This task was chosen because it is known to engage frontal, sensory-motor, temporal, and parietal brain regions known to demonstrate both resilience and plasticity in their functional organization for bilingualism and sign language exposure (Mechelli et al., 2004; Abutalebi et al., 2008; Kovelman et al., 2009; Emmorey et al., 2011). Participants' brain activity was recorded with functional Near Infrared Spectroscopy (fNIRS) while they completed the task. fNIRS is well suited for studies of higher cognition because it has both good temporal sampling (10–50 Hz) and good spatial resolution (~3 cm depth) without being in an enclosed structure (Huppert et al., 2009; Shalinsky et al., 2009). Hence, the two critical innovations of the study is the comparison between deaf and hearing bimodal bilinguals and the use of fNIRS technology to study sign language production in its ecologically natural form, that is, when participants are upright and using their arms and hands freely.

## Materials and methods

### Participants

Three (3) participant groups included 17 hearing, native English monolinguals with no knowledge of signed language, 7 deaf native ASL signers, and 5 *hearing* native ASL-English bimodal bilinguals from birth (see Assessments below and Table 1). Five of the 7 deaf native ASL signers were congenitally deaf while two lost hearing before age 1. Five of the seven deaf native ASL signers first learned ASL from deaf family members, including parents and siblings. For the remaining two participants from this group, the primary source of ASL was preschool for the deaf between the ages of 1–3. The 7 deaf native ASL signers had been exposed to English as a second language at school and had overwhelmingly rich ASL daily language exposure, use, and maintenance throughout adulthood. All 7 deaf native ASL signers considered themselves to be functionally dominant ASL users. All participants were right handed, using Edinburgh Handedness Inventory (Oldfield, 1971). The participants in this study were also the participants examined in a separate study (Kovelman et al., 2009).

**Table 1:** **Participant groups' ages and language background and participant information**.

**Group**	**Mean age (range)**	**Parents' native language(s)**	**Age of language exposure**		**Mean performance on language proficiency tasks**	
			**Eng**	**ASL**	**Eng**	**ASL**
English monolinguals *n* = 17 (10 female)	19 (18–25)	English only	Birth		96%	
Deaf ASL signers *n* = 7 (4 female)	26 (19–42)	ASL English both		Birth–4 years		100%
ASL-English bilinguals *n* = 5 (4 female)	24 (16–32)	ASL English both	Birth	Birth	96%	98%

#### Participant assessments

All participants completed a previously standardized and published Language Background and Use Questionnaire that allowed us to assess their monolingual and/or bilingual language acquisition history and language use. All bilingual participants reported early (before age 5) and systematic exposure to both languages. All participants also reported continual use of the two languages. Participants also completed a previously published Language Competence/Expressive Proficiency (LCEP) test (for details on test administration and scoring see Kovelman et al., 2008a,b,c; Berens et al., 2013). LCEP builds upon measures of sign language competence, originally established for the study of Nicaragual Sign Language (Senghas and Kegl, 1994), and was expanded for use with other sign and spoken languages (Petitto et al., 2001a; Schembri and Johnston, 2004; Jasinska and Petitto, 2013). All participants were required to score >80% on the LCEP in each of their native languages to be eligible for this study—thus demonstrating comparably high language competence in each of their languages. The study was approved by the Internal Ethical Review Board of Dartmouth College (Hanover, New Hampshire, USA). All participants were students and professionals in a local college township area.

### Brain imaging task

An ASL sign repetition task was used as our imaging task. This is a standard task in sign modeled after the classic word repetition task. The task allows for the study of sign production in both native signers and individuals naïve to sign languages (cf. Petitto et al., 2000). The participants watched a video recording of a native ASL signer producing ASL Real signs and Pseudosigns, one at a time. Pseudosigns obey the linguistic rules for phonological and lexical formation in ASL but do not exist in ASL, and are analogous to pseudowords in English. After each sign or pseudosign presentation, the participants repeated the sign or pseudosign. This was a block design with stimuli blocked by condition [sign/pseudosign, 12 blocks per condition, 5 trials per block (30 s blocks), 6 s per trial].

Real signs were meaningful, single-handed, high-frequency nouns. These were based on the corpus of signs commonly acquired by young ASL speakers before the age of 6 (Petitto et al., 2001a; Holowka et al., 2002; Petitto and Kovelman, 2003). Pseudosigns consisted of hand movements that were meaningless, sign-phonetic units that were syllabically organized into possible but non-existing short syllable strings. Like spoken languages, all words in signed languages are formed from a finite set of meaningless units, called phonetic units (for example, an unmarked, frequent phonetic unit in sign involves a clenched fist with an extended thumb). To ensure ecological validity of the pseudoword task, we used real but meaningless phonetic units, including those documented in ASL-exposed infants during manual babbling (Petitto and Marentette, 1991; Petitto et al., 2001b). These phonetic units were further organized into syllables (e.g., specific hand shapes organized into temporally constrained, rhythmic-movement-nonmovement alternations). Rigorous psycholinguistic experimental procedures were used to pilot the stimuli to ensure that they indeed contained true meaningless phonetic and syllabic units. For more details about these methods and the stimuli please see Petitto et al. (2000); for video demonstration of the stimuli please see supplementary PNAS methods or visit http://petitto.gallaudet.edu/

### Data acquisition, anatomical coregistration, and fNIRS signal analysis

**Figure 1:**
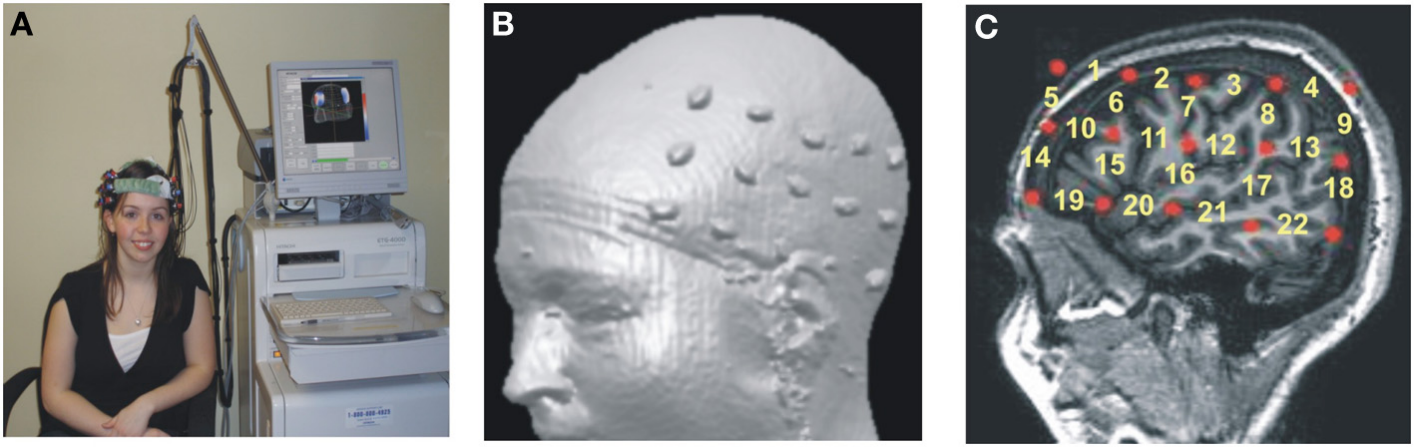
**Hitachi ETG-4000 Imaging System MRI Neuroanatomical Co-Registration. (A)** Participant with a Hitachi 48-channel ETG-4000. Optodes in place and ready for data acquisition. The 3 × 5 optode arrays were positioned on participants' heads using rigorous anatomical localization measures including 10 × 20 system and MRI coregistration. **(B)** MRI co-registration was conducted by having nine healthy adults wear two 3 × 5 arrays with vitamin-E capsules in place of the optodes in MRI. **(C)** Anatomical MRI images were used to identify the location of optodes (Vitamin E capsules) with respect to underlying brain structures (sagittal section shown here). Location of the fNIRS channels numbered in yellow.

The accurate neuroanatomical placement of fNIRS probes and the confirmation of ROIs (regions of interest) were achieved by using the 10–20 system (Jurcak et al., 2007; Shalinsky et al., 2009). The 10–20 anchor points were established using published neuroanatomical landmarks for the 10–20 system (Koessler et al., 2009) as well as by obtaining MRI scans for a subset of participants (*n* = 9; Figures 1B,C). Stereotactic localization of the probe array was further confirmed for each participant by using a Fasttrak spatial detection system (Polhemus, Colchester, VT). During fNIRS scanning, participants wore a soft headband holding the fNIRS probes while positioned comfortably in a reclining chair, with the fiber optics hanging loosely without making contact with the body or chair (Figure 1A).

fNIRS data was recorded at 10 Hz and high-pass filtered at 0.5 Hz to remove physiological noise (especially heart rate). The modified Beer-Lambert equation (mBL; Delpy et al., 1988) was used to convert wavelength data to oxy- and deoxy-hemoglobin concentrations. The application of the mBL was conducted in two steps. Under the assumption of constant scattering over the path length, the attenuation for each wavelength (A_(*t*)_) was calculated by comparing the optical density of light intensity during the task (I_*task*_) to the calculated baseline of the signal (I_*baseline*_). We then used these A values for each wavelength and sampled time point (*t*) to solve the modified Beer-Lambert equation (Equation 1; for more information see Huppert et al., 2009; Shalinsky et al., 2009).

(1) $$\Delta {\text{A}}_{\lambda (t)}\;=\log_{10}\left(\frac{{\text{I}}_{task}}{{\text{I}}_{baseline}}\right)$$

(2) $$\left(\frac{\Delta {\text{A}}_{\lambda 1}(t)}{\Delta {\text{A}}_{\lambda 2}(t)}\right)=\left[\begin{array}{cc} \varepsilon_{deoxy}^{\lambda 1} & \varepsilon_{oxy}^{\lambda 1}\\ \varepsilon_{deoxy}^{\lambda 2} & \varepsilon_{oxy}^{\lambda 2} \end{array}\right]\left(\begin{array}{c} C_{deoxy_{\left(t\right)}}\\ C_{oxy_{\left(t\right)}} \end{array}\right)$$

λ_1_ε_*deoxy*_, λ_1_ε_*deoxy*_, λ_2_ε _*deoxy*_, and λ_2_ε_*oxy*_ are the constants for the extinction coefficients that measure the fraction of light lost to absorption per unit concentration distance in the tissue (Equation 2). The resultant C_*deoxy*_ and C_*oxy*_ values are the concentrations of deoxygenated and oxygenated hemoglobin for each *t*.

C_*deoxy*_ and C_*oxy*_ values for each channel were then plotted and inspected. The maximum positive or negative peak values (for oxy and deoxy, respectively) were determined for each channel from 5 s after the onset of the trial until the end of the trial. To compute the baseline values of oxy- and deoxy-hemoglobin concentrations, we took the mean of the 5 s-segment leading up to the start of the trial (for more detail on pre and post-processing analytical options for fNIRS, see Huppert et al., 2009; Shalinsky et al., 2009; Gervain et al., 2011; Tak and Ye, 2014). These values were then used in the statistical analysis.

**Figure 2:**
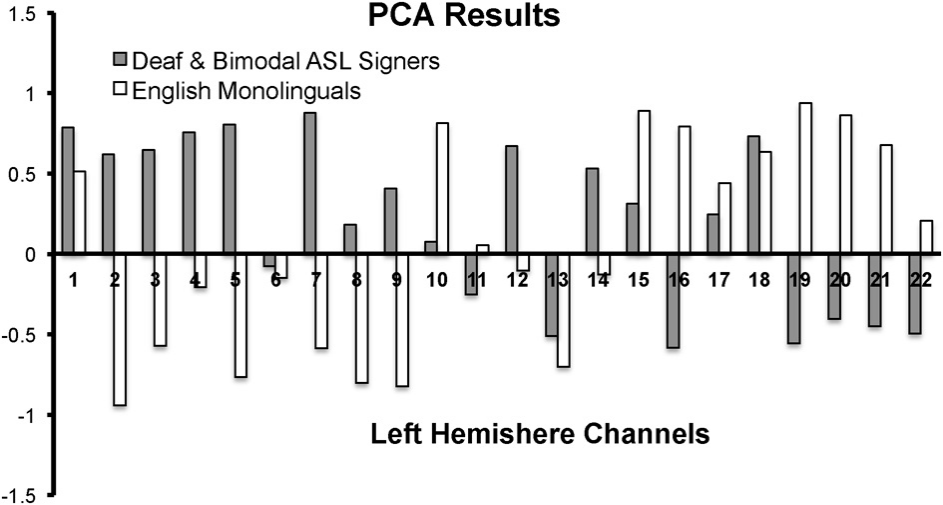
**PCA results for the first component for the left hemisphere channels during the Real Sign condition**. The first component accounted for 41% variance in English monolinguals and 30% variance in Deaf and Bimodal ASL signers.

First, we conducted a principle component analysis (PCA) to explore any differences in the pattern of brain activity between signers (Deaf and Bimodal) and non-signers (monolingual English speakers). Potential differences in the left hemisphere typically associated with language processes in speech and in sign were of critical interest (Petitto et al., 2000), and hence the PCA analyses were limited to the left hemisphere's channels (Figure 2). Second, we used anatomical information from MRI probe co-registration (Figure 1), in combination with present and prior PCA findings for sign and spoken languages (Kovelman et al., 2009), to reduce the 22 channels per hemisphere to 7 regions of interest for each hemisphere.

Finally, to examine differences between bimodal and Deaf signers we conducted repeated measures ANOVAs with follow-up Mann-Whitney *t*-test for non-related samples. The analyses were conducted for each hemisphere separately, while using two types of comparisons: one with Real and Pseudosign treated as separate factors, and one with both conditions averaged to optimize the study's power given the low number of participants.

## Results

### Behavioral results

Native ASL signers scored participants' accuracy on the basis of the phonological constituents of sign, hand-shape and hand-movement, for each sign and pseudosign (Petitto et al., 2000). Both groups received training with the task, as it was important that the groups be as accurate as possible with their hand movements, while maintaining good head-posture. As a result, all groups showed a relatively high task performance (Table 2). We used a 3 × 2 mixed-measures ANOVA to compare language groups (English monolinguals, bimodal bilinguals, deaf ASL signers; between group factor) across two language conditions (Signs vs. Pseudosigns; within group factor) and found significant effects of group [*F*_(2, 24)_ = 6.7, *p* = 0.005], task [*F*_(1, 24)_ = 8.9, *p* = 0.006] and the interaction [*F*_(2, 24)_ = 3.5, *p* = 0.04]. *Post-hoc* comparisons revealed a significantly better performance in Deaf Signers relative to English monolinguals (Tukey's HSD, *p* < 0.05), but no significant differences between the two hearing groups (*p* > 0.05). Additional follow-up paired *t*-test comparisons revealed that Deaf signers [*t*_(6)_ = 3.6, *p* = 0.01] and Bimodal Bilinguals [*t*_(5)_ = 2.8, *p* = 0.07], but *not* English monolinguals [*t*_(16)_ = −1.9, *p* = 0.85], were more accurate in repeating the Real signs than Pseudosigns (Table 2).

**Table 2:** **Participants' task performance, Mean (Standard Deviation) behavioral scores measured in percent correct for the sign repetition task by language group**.

**Group**	**Real signs**	**Pseudosigns**
Deaf ASL signers	99 (0.01)	93 (0.04)
Bimodal bilinguals	98 (0.03)	90 (0.09)
English monolinguals	89 (0.06)	89 (0.07)

### fNIRS results

#### PCA analysis

In native ASL signers, PCA yielded a first component that explained 41% of the variance in English monolinguals and 30% variance in native signers (across Deaf and Bimodal signers). The pattern of the positive/negative loading for the first component was nearly the opposite between signers and non-signers (Figure 2). The negative loadings in signers (and positive in non-signers) appeared to span superior frontal and sensory-motor regions. The positive loadings for signers (and negative for non-signers) appeared to span inferior frontal and occipito-temporal regions.

The contrasting pattern of PCA loadings suggested that the two groups treated the task differently, possibly due to the *linguistic* vs. *non-linguistic* nature of sign processing in Deaf and bimodal signers, relative to English monolinguals (Petitto et al., 2000; Emmorey et al., 2005). Hence, all of our subsequent analyses only included native signers because our primary target was *language* processing in bilinguals and monolinguals. Moreover, the differences or similarities in mental processes between signers and non-signers might not necessarily be reflected by differences in the strength of activation in regions such as IFG, which are thought to support both language (Poeppel et al., 2012) and movement imitation (Kilner et al., 2009).

#### Brain regions of interest

The study aimed to bridge findings between prior bilingual research on spoken languages with present findings for sign language. Hence, to ensure that the results were maximally comparable across past and present findings, the channel grouping into specific regions was governed by previously published grouping for this same set of participants (same group of participants but a comparison of English language task between bimodal bilinguals to English monolinguals; Kovelman et al., 2009), MRI probe coregistration (Figure 1) and the PCA results. The channels were grouped into seven regions as follows. **(i) Inferior frontal/anterior STG** (BA 44/45 and 38/22, channels 15, 19, 20, overlapping with the classic Broca's area). Please note that we recognize that IFG and anterior STG are two distinct anatomical areas. However, since channels overlaying these areas consistently clustered together in every PCA analysis (including sign repetition, see below), we combine these areas into one data- and prediction-driven functional/anatomical ROI. **(ii) Dorsolateral prefrontal cortex (DLPFC)** (BA 9/46, channels 10 and 14), **(iii) superior frontal** (BA 9/10, channels 1, 5), **(iv) posterior temporal** (BA 21/22, channel 17, overlapping with classic Wernicke's area), **(v) parietal** (including inferior and superior parietal regions, BA 7/39/40; channels 4, 8, 9, 13), **(vi) sensory-motor** (BA 1–6, channel 7), and **(vii) premotor** (BA 9/6; channels 6 and 11). Average and standard deviations of signal changes for each ROI, each group and each experimental condition are presented in Table 3.

**Table 3:** **Participants' brain activation, Mean (Standard Deviation) brain activation as measured in fnirs percent signal intensity during sign repetition task by condition and brain region**.

**Group**	**Condition**	**IFG/anterior STG**	**Posterior temporal**	**DLPFC**	**Superior frontal**	**Parietal**	**Posterior frontal/motor**	**Sensory-motor**
		**BA 44/45 and 21**	**BA 21/22**	**BA 9/46**				**BA 1–5**
**A. LEFT HEMISPHERE**								
English monolinguals	Real sign	0.42 (0.27)	0.28 (0.17)	0.38 (0.16)	0.31 (0.16)	0.42 (0.18)	0.35 (0.19)	0.42 (0.26)
	Non-sign	0.50 (0.21)	0.32 (0.17)	0.46 (0.22)	0.33 (0.23)	0.32 (0.20)	0.40 (0.20)	0.47 (0.27)
Deaf ASL signers	Real sign	0.42 (0.24)	0.22 (0.07)	0.37 (0.16)	0.37 (0.27)	0.35 (0.13)	0.39 (0.25)	0.37 (0.31)
	Non-sign	0.39 (0.19)	0.43 (0.29)	0.39 (0.15)	0.43 (0.18)	0.44 (0.17)	0.45 (0.16)	0.52 (0.25)
ASL-English bilinguals	Real sign	0.52 (0.28)	0.20 (0.06)	0.31 (0.19)	0.28 (0.08)	0.22 (0.09)	0.37 (0.13)	0.41 (0.12)
	Non-sign	0.46 (0.17)	0.24 (0.17)	0.23(0.14)	0.25 (0.12)	0.29 (0.12)	0.30 (0.20)	0.36 (0.22)
**B. RIGHT HEMISPHERE**								
English monolinguals	Real sign	0.36 (0.16)	0.26 (0.19)	0.38 (0.24)	0.35 (0.19)	0.42 (0.22)	0.36 (0.22)	0.43 (0.19)
	Non-sign	0.37 (0.25)	0.26 (0.23)	0.33 (0.18)	0.35 (0.26)	0.35 (0.18)	0.42 (0.21)	0.41 (0.27)
Deaf ASL signers	Real sign	0.39 (0.20)	0.34 (0.21)	0.43 (0.17)	0.46 (0.26)	0.40 (0.15)	0.29 (0.19)	0.32 (0.22)
	Non-sign	0.44 (0.27)	0.30 (0.18)	0.42 (0.25)	0.43 (0.25)	0.41 (0.14)	0.26 (0.13)	0.36 (0.21)
ASL-English bilinguals	Real sign	0.51 (0.19)	0.28 (0.14)	0.39 (0.27)	0.40 (0.27)	0.31 (0.16)	0.39 (0.16)	0.55 (0.27)
	Non-sign	0.45 (0.19)	0.39 (0.21)	0.28 (0.15)	0.36 (0.14)	0.41 (0.19)	0.30 (0.15)	0.41 (0.22)

#### Brain bases of sign repetition in bimodal bilinguals and deaf signers

**Figure 3:**
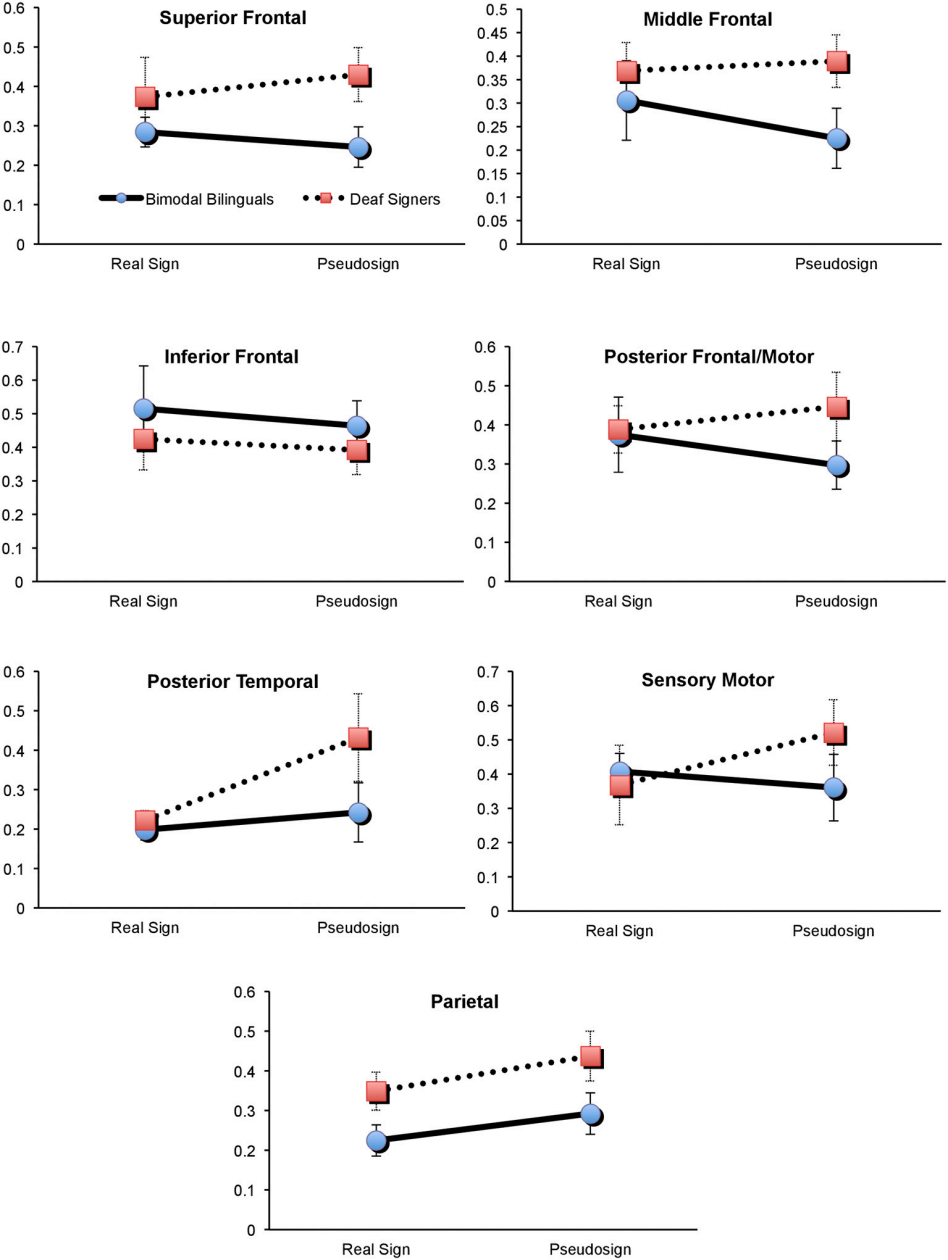
**Brain activation in Deaf and Bimodal ASL users during Sign and Pseudosign conditions for all left hemisphere brain regions**. Error bars represent standard error of the mean.

First, we explored potential interaction between participants' brain activation and the type of experimental condition (sign vs. pseudosign) using a 2 × 2 × 7 mixed measures ANOVA (groups × task × all brain regions), conducted separately for each hemisphere. *Left hemisphere* analyses revealed a marginally-significant task × group interaction [*F*_(1, 60)_ = 3.7, *p* = 0.08], and a marginally-significant main effect of group [*F*_(1, 10)_ = 4.2, *p* = 0.06]. There were no significant main effects of task (*p* = 0.36) or brain regions (*p* = 0.13). To identify the source of group × task interaction, we conducted two 2 × 7 mixed measures ANOVAs (groups × regions), one for Real and one for Pseudosign conditions. Group differences remained significant only during the Pseudosign condition [*F*_(1, 10)_ = 9.7, *p* = 0.01], with Deaf signers showing overall greater activation during Pseudosign condition, especially in left MFG [*t*_(10)_ = −2.2, *p* = 0.03] and SFG [*t*_(10)_ = −1.9, *p* = 0.07] regions. In sum, while the two groups of participants showed overall similar levels of activation during the Real Sign condition, this activation increased in Deaf signers during the Pseudosign condition, but decreased or stayed the same in bimodal bilinguals (Figure 3). *Right hemisphere* analyses did not yield any significant effects.

Second, we explored the possible presence of overall activation differences between bimodal and Deaf signers, differences that may span both Real and Pseudosign conditions. For this comparison we conducted two 2 × 7 mixed measures ANOVAs (groups × regions), with activation averaged across Real and Pseudosign conditions, separately for each hemisphere. *Left hemisphere* analyses revealed marginally significant group differences [*F*_(1, 10)_ = 4.2, *p* = 0.06] and no main effects of region (*p* = 0.13) or interactions (*p* = 0.66). Bimodal bilinguals showed lower overall levels of left hemisphere activation relative to Deaf signers, the differences reached significance in the left parietal region [*t*_(10)_ = −2.0, *p* = 0.03; Figure 3]. *Right hemisphere* analyses did not yield any significant effects. The results are also summarized in Figure 3.

## Discussion

The study investigated the extent of resiliency (“fixed” or stable brain structures, systems, and their functions) and plasticity (neural plasticity of brain structures, systems, and their functions) of human language organization in the brain. We used fNIRS imaging to study native hearing ASL-English bimodal bilinguals as compared to native Deaf ASL signers to investigate the nature of bilingual brain organization for language.

Relative to spoken language monolinguals, unimodal bilinguals have been shown to demonstrate different levels of brain activation across left frontal, sensory-motor, and temporo-parietal regions of the brain (Kovelman et al., 2009; Jasinska and Petitto, 2013). Bilingual theories suggest that the need to resolve competing sensory-motor and lexical representations drives many of these neurodevelopmental changes (Abutalebi et al., 2008; Garbin et al., 2010). It is possible that such effects are reduced in bimodal bilinguals because their two languages are less likely to compete for sensory-motor production and encoding (Emmorey et al., 2008). It has been previously found that bimodal bilinguals show greater activation in left temporo-parietal regions during language switching relative to their unimodal use of English or to English monolinguals (Kovelman et al., 2009), but it remained unclear if this was due to bilingualism or exposure to sign languages, which places special demands on these regions (Newman et al., 2002). The present findings revealed multiple ways in which the left hemisphere brain activation in bimodal bilinguals differed from Deaf signers, including differences in the left parietal regions. These data suggest that there is indeed an interaction between bilingual and sign language exposure on the bimodal bilingual's language organization in the brain.

The behavioral data revealed that during the Sign Repetition task, the two sign-exposed groups had comparable language competence and experimental task performance. In contrast, the neuroimaging data revealed information that behavioral data alone could not. First, we found that bilingual exposure to sign and speech had a broad spectrum effect on the bilingual's brain bases of language processing throughout the left but not the right hemisphere regions. Specifically, pseudowords are thought to incur greater effort on parts of the language mechanisms (and their neural correlates) that participate in word search and retrieval (Zatorre et al., 1996; Petitto et al., 2000; Xiao et al., 2005; Koenigs et al., 2011; Perrachione et al., under review). Yet, the findings suggest that Deaf signers showed a stronger and more widespread increase in brain activation during pseudo-sign relative to Real sign conditions (Figure 3). As a result, there was a significant difference in overall activation between the two groups during the pseudo-sign condition.

The interaction effect for the Real sign vs. Pseudosign condition was especially pronounced in the MFG and SFG frontal regions of the left hemisphere. Specifically, Deaf signers showed significantly greater activation in the two regions relative to bilingual bimodal signers. These left middle and superior frontal regions are typically associated with verbal working memory and other aspects of executive functioning that support overall language processing (Adleman et al., 2002). Repetition of a pseudoword requires one to perceive, encode, maintain, and repeat an unfamiliar linguistic unit that cannot be retrieved from memory (Petitto et al., 2000). Hence, it is possible that Deaf signers showed greater activation in these regions because of the increased working memory load for unfamiliar words. For bilingual participants, these regions also form the frontal lobe aspect of the bilingual switching mechanism (Abutalebi et al., 2008; Kovelman et al., 2008c). One possibility is that dual language switching experiences have enhanced the functionality of these frontal regions in bilinguals, such that both familiar and unfamiliar words exert similar amounts of effort on the part of these frontal regions.

Another possibility is that the bilingual brain is better adapted to encountering and learning new words. Prior bilingual studies have shown that bilinguals tend to have lower vocabularies in each of their languages relative to monolinguals, likely due to varied types of language experiences across the bilinguals' two languages (Petitto et al., 2001a). Hence, encountering and encoding new words might be a more common-place experience for bilinguals, resulting in lower effort on the part of the frontal lobe regions for this task. Overall, the increase of bilateral frontal lobe activation is typically found during bilinguals' non-native language processing (Kim et al., 1997; Wartenburger et al., 2003) or language switching (e.g., Hernandez et al., 2001; Abutalebi et al., 2008). Yet, less is known about bilingual brain functioning during the regular unilingual use of their first language. Our data suggests that such bilingual brain activation might be reduced relative to monolinguals, especially during more demanding language tasks, such as processing of pseudowords.

Prior research comparing bimodal bilinguals to English monolinguals has shown greater activation in the left temporo-parietal regions in bimodal bilinguals (Kovelman et al., 2009). Yet, it remained unclear if this was due to the bilingual exposure in general or sign language exposure in particular, given the critical importance of the parietal regions for native-like linguistic processing of sign languages (Neville et al., 1997; Corina et al., 1999; Petitto et al., 2000; Corina and McBurney, 2001; Newman et al., 2002; Emmorey et al., 2007, 2011). The present findings suggest that hearing native ASL-English bimodal bilinguals had reduced neural recruitment of the left parietal region relative to the deaf native ASL users. The findings are thus parallel to spoken language findings in bimodal bilinguals: the two languages of bimodal bilinguals (sign and speech) show *less* activation in left temporo-parietal regions during the unimodal use of only one of the two languages relative to their use of two languages simultaneously or in rapid alternation. Hence, we suggest that this finding is *not* explained by sensory differences (hearing vs. deaf) or because the bimodal bilingual's languages exist in two different modalities thereby minimizing sensory-motor competition (Hyp. 1). Instead, we interpret our results as supporting Hypothesis 2. We suggest that the neural demands/impact of early *bilingual* language exposure may influence the functional organization of the left parietal brain region in ways that, in turn, offer a possible explanation of the observed differences between our bimodal bilinguals and our native sign language users.

Neuroimaging studies have found that increased expertise in a higher cognitive function *lowers* activation in brain regions dedicated to that function (Xue and Poldrack, 2007; Chein and Schneider, 2012). Is it possible that *lower* activation in left parietal and other regions of the bimodal bilinguals reflects a more efficient manner of language processing? Our previous study with this exact group of bimodal bilinguals and English monolinguals revealed that bimodal bilinguals showed *heightened* activation in their left temporo-parietal regions *depending on the bilingual language processing context*. Bimodal bilinguals showed heightened activation in left temporo-parietal regions during bilingual language switching tasks relative to non-switching language conditions (Kovelman et al., 2009). The present findings show that bimodal bilinguals had lower activation in this general region during the processing of signs, as compared to Deaf signers. Such converging evidence suggests that bilingual exposure impacts language organization in parietal and other left hemisphere regions in bimodal bilinguals as compared to functionally-monolingual users of sign or speech.

This study used the case of bimodal bilingualism to address the field's core question of whether sensory-motor competition between two languages is necessary to change language organization in the brain of a bilingual individual (Bialystok, 2001; Abutalebi et al., 2008; Emmorey et al., 2008; Kovelman et al., 2009). The study found both focal (parietal) and broad-spectrum differences (especially during pseudo-sign) in left-hemisphere activation between bimodal bilinguals and Deaf signers. One possible explanation is that the data confirms hypothesis 2: *the hearing and signing bimodal bilingual experience does impact the brain and that the impact of bilingualism extends beyond the sensory-motor competition*, and reflects an accommodation for learning and using two complex linguistic systems. It is possible that the extensive bilingual exercise of left hemisphere language regions may have improved their functionality in bilinguals. Hence, bimodal bilinguals showed an overall lowering of levels of activation throughout the left hemisphere relative to Deaf signers and did not show an increase in these regions' activity even as the task demands increased.

Another possible explanation for the findings is that they stem not from the *hearing*, but from the *deaf* bimodal bilingual experiences. Even though the Deaf participants in the study were functionally monolingual (predominantly relied on ASL for their daily communication), research has recently found that Deaf signers have greater left hemisphere volume in frontal and motor regions relative to hearing signers (Allen et al., 2013). This might be due to deaf individuals' accommodation for the complex task of mastering spoken language(s) via the visual modality (Allen et al., 2013). Thus, on the one hand, the study confirms that bilingual exposure changes the brain. Yet, on the other hand, the study does not provide strong adjudicating evidence of whether it was the hearing or the deaf bimodal language learning experience that best explains the findings. Future studies will need to simultaneously consider both functional and neuroanatomical evidence to better understand the impact of bimodal language experience on the language organization in the brain.

Finally, the present findings found nearly opposing PCA brain imaging results for signers and non-signers during the sign repetition task, even though non-signers were able to achieve nearly 90% accuracy in repeating the signs. The results suggest that brain regions cluster or work together differently for signers vs. non-signers during an ASL language task. Prior imaging studies with other brain imaging modalities (e.g., fMRI, PET, ERP) have consistently demonstrated linguistic vs. non-linguistic patterns of sign processing by the brain in deaf signers relative to hearing non-signers (Hickok et al., 1996; Neville et al., 1997; MacSweeney et al., 2002a,b; Corina et al., 2003; Emmorey et al., 2007), including the study that used the exact same imaging task (Petitto et al., 2000). Therefore, we hope to have imparted to our readers confidence in the effectiveness of the fNIRS imaging method for research on questions involving neural recruitment during human language processing.

One of the study's caveats is the low number of participants and hence, the inability to achieve higher statistical thresholds or control for multiple comparisons. Native signers are becoming an increasingly low incidence population and thus, very challenging to recruit and to study—especially involving neuroimaging studies in light of the dramatic rise in the availability of auditory augmentation technologies (e.g., cochlear implants). The other caveat is the relatively large regions of the brain covered by individual fNIRS channel measurements. Nonetheless, the present findings provide powerful corroborating evidence with other studies of signers, which had used larger samples and technologies with better neuroanatomical spatial resolution (e.g., Corina et al., 2003; Emmorey et al., 2007). Together, all such studies contribute to the growing volume of knowledge that both differences in early life experience and variation in cross-modal language processing can have on the neural systems and processing of human language.

## Conclusion

Through a novel view provided from individuals who are bilingual across two different language modalities (signed and spoken), we have observed the brain's remarkable neural *plasticity* of brain regions underlying human language processing. In particular, we gained insight into the multiple and varied conditions by which particular brain sites may be recruited. The parietal regions that have been viewed as selectively sensitive to sign language phonology and syntax (Newman et al., 2002), as well as language switching in bimodal bilingual signers (Kovelman et al., 2009), also showed a modulation based on the bilingualism status of the language user. The present findings also provide support for the use of fNIRS technology in studying human brain function. Taken together, the findings expand our knowledge of the “signing” and the “bilingual brain,” and provide new insights into the resilience and plasticity of our brain's remarkable capacity for human language.

### Conflict of interest statement

The authors declare that the research was conducted in the absence of any commercial or financial relationships that could be construed as a potential conflict of interest.

## References

[B1] Abutalebi J. Annoni J. M. Zimine I. Pegna A. J. Seghier M. L. Lee-Jahnke H. (2008). Language control and lexical competition in bilinguals: an event-related fMRI study. Cereb. Cortex 18, 1496–1505 10.1093/cercor/bhm182 17947346

[B2] Abutalebi J. Cappa S. F. Perani D. (2001). The bilingual brain as revealed by functional neuroimaging. Bilingualism Lang. Cognit. 4, 179–190 10.1017/S136672890100027X

[B3] Adleman N. E. Menon V. Blasey C. M. White C. D. Warsofsky I. S. Glover G. H. (2002). A developmental fMRI study of the Stroop color-word task. Neuroimage 16, 61–75 10.1006/nimg.2001.1046 11969318

[B4] Allen J. S. Emmorey K. Bruss J. Damasio H. (2013). Neuroanatomical differences in visual, motor, and language cortices between congenitally deaf signers, hearing signers, and hearing non-signers. Front. Neuroanat. 7:26 10.3389/fnana.2013.00026 23935567 PMC3731534

[B79] Berens M. S. Kovelman I. Petitto L. A. (2013). Should bilingual children learn reading in two languages at the same time or in sequence? Biling Res J. 36, 35–60 10.1080/15235882.2013.779618 23794952 PMC3685861

[B7] Bialystok E. (2001). Bilingualism in Development: Language, Literacy, and Cognition. New York, NY: Cambridge University Press 10.1017/CBO9780511605963

[B8] Bialystok E. (2008). Bilingualism: the good, the bad, and the indifferent. Bilingualism Lang. Cognit. 12, 3–11 10.1017/S1366728908003477

[B9] Bialystok E. Craik F. I. M. Luk G. (2012). Bilingualism: consequences for mind and brain. Trends Cogn. Sci. (Regul. Ed) 16, 240–250 10.1016/j.tics.2012.03.001 22464592 PMC3322418

[B10] Blumenfeld H. K. Marian V. (2013). Parallel language activation and cognitive control during spoken word recognition in bilinguals. J. Cogn. Psychol. 25, 547–567 10.1080/20445911.2013.812093 24244842 PMC3827904

[B13] Chein J. M. Schneider W. (2012). The brain's learning and control architecture. Curr. Dir. Psychol. Sci. 21, 78–84 10.1177/0963721411434977

[B14] Corina D. P. McBurney S. L. (2001). The neural representation of language in users of American Sign Language. J. Commun. Disord. 34, 455–471 10.1016/S0021-9924(01)00063-6 11725858

[B15] Corina D. P. McBurney S. L. Dodrill C. Hinshaw K. Brinkley J. Ojemann G. (1999). Functional roles of Broca's area and SMG: evidence from cortical stimulation mapping in a deaf signer. Neuroimage 10, 570–581 10.1006/nimg.1999.0499 10547334

[B16] Corina D. P. San Jose-Robertson L. Guillemin A. High J. Braun A. R. (2003). Language lateralization in a bimanual language. J. Cogn. Neurosci. 15, 718–730 10.1162/jocn.2003.15.5.718 12965045

[B18] Delpy D. T. Cope M. van der Zee P. Arridge S. Wray S. Wyatt J. (1988). Estimation of optical pathlength through tissue from direct time of flight measurement. Phys. Med. Biol. 33, 1433–1442 10.1088/0031-9155/33/12/008 3237772

[B20] Emmorey K. Grabowski T. McCullough S. Ponto L. L. B. Hichwa R. D. Damasio H. (2005). The neural correlates of spatial language in English and American Sign Language: a PET study with hearing bilinguals. Neuroimage 24, 832–840 10.1016/j.neuroimage.2004.10.008 15652318

[B21] Emmorey K. Luk G. Pyers J. E. Bialystok E. (2008). The source of enhanced cognitive control in bilinguals: evidence from bimodal bilinguals. Psychol. Sci. 19, 1201–1206 10.1111/j.1467-9280.2008.02224.x 19121123 PMC2677184

[B22] Emmorey K. McCullough S. (2009). The bimodal bilingual brain: effects of sign language experience. Brain Lang. 109, 124–132 10.1016/j.bandl.2008.03.005 18471869 PMC2680472

[B23] Emmorey K. Mehta S. Grabowski T. J. (2007). The neural correlates of sign versus word production. Neuroimage 36, 202–208 10.1016/j.neuroimage.2007.02.040 17407824 PMC1987366

[B24] Emmorey K. Xu J. Braun A. (2011). Neural responses to meaningless pseudosigns: evidence for sign-based phonetic processing in superior temporal cortex. Brain Lang. 117, 34–38 10.1016/j.bandl.2010.10.003 21094525 PMC3075318

[B25] Finkbeiner M. Gollan T. H. Caramazza A. (2006). Lexical access in bilingual speakers: what's the (hard) problem? Bilingualism Lang. Cognit. 9, 153–166 10.1017/S1366728906002501

[B27] Garbin G. Sanjuan A. Forn C. Bustamante J. C. Rodriguez-Pujadas A. Belloch V. (2010). Bridging language and attention: brain basis of the impact of bilingualism on cognitive control. Neuroimage 53, 1272–1278 10.1016/j.neuroimage.2010.05.078 20558314

[B28] Gervain J. Mehler J. Werker J. F. Nelson C. A. Csibra G. Lloyd-Fox S. (2011). Near-infrared spectroscopy: a report from the McDonnell infant methodology consortium. Dev. Cogn. Neurosci. 1, 22–46 10.1016/j.dcn.2010.07.004 22436417 PMC6987576

[B29] Grosjean F. (1989). Neurolinguists, beware! The bilingual is not two monolinguals in one person. Brain Lang. 36, 3–15 10.1016/0093-934X(89)90048-5 2465057

[B30] Hernandez A. E. Dapretto M. Mazziotta J. Bookheimer S. (2001). Language switching and language representation in Spanish-English bilinguals: an fMRI study. Neuroimage 14, 510–520 10.1006/nimg.2001.0810 11467923

[B31] Hickok G. Bellugi U. Klima E. S. (1996). The neurobiology of sign language and its implications for the neural basis of language. Nature 381, 699–702 10.1038/381699a0 8649515

[B32] Holowka S. Brosseau−Lapré F. Petitto L. A. (2002). Semantic and conceptual knowledge underlying bilingual babies' first signs and words. Lang. Learn. 52, 205–262 10.1111/0023-8333.00184

[B33] Huppert T. J. Diamond S. G. Franceschini M. A. Boas D. A. (2009). HomER: a review of time-series analysis methods for near-infrared spectroscopy of the brain. Appl. Opt. 48, D280–D298 10.1364/AO.48.00D280 19340120 PMC2761652

[B34] Jasinska K. K. Petitto L. A. (2013). How age of bilingual exposure can change the neural systems for language in the developing brain: a functional near infrared spectroscopy investigation of syntactic processing in monolingual and bilingual children. Dev. Cogn. Neurosci 6, 87–101 10.1016/j.dcn.2013.06.005 23974273 PMC6987800

[B37] Jurcak V. Tsuzuki D. Dan I. (2007). 10/20, 10/10, and 10/5 systems revisited: their validity as relative head-surface-based positioning systems. Neuroimage 34, 1600–1611 10.1016/j.neuroimage.2006.09.024 17207640

[B38] Kilner J. M. Neal A. Weiskopf N. Friston K. J. Frith C. D. (2009). Evidence of mirror neurons in human inferior frontal gyrus. J. Neurosci. 29, 10153–10159 10.1523/JNEUROSCI.2668-09.2009 19675249 PMC2788150

[B39] Kim K. H. S. Relkin N. R. Lee K.-M. Hirsch J. (1997). Distinct cortical areas associated with native and second languages. Nature 388, 171–174 10.1038/40623 9217156

[B41] Klein D. Mok K. Chen J. K. Watkins K. E. (2014). Age of language learning shapes brain structure: a cortical thickness study of bilingual and monolingual individuals. Brain Lang. 131, 20–24 10.1016/j.bandl.2013.05.014 23819901

[B42] Koenigs M. Acheson D. J. Barbey A. K. Solomon J. Postle B. R. Grafman J. (2011). Areas of left perisylvian cortex mediate auditory-verbal short-term memory. Neuropsychologia 49, 3612–3619 10.1016/j.neuropsychologia.2011.09.013 21945329 PMC3209761

[B43] Koessler L. Maillard L. Benhadid A. Vignal J. P. Felblinger J. Vespignani H. (2009). Automated cortical projection of EEG sensors: anatomical correlation via the international 10-10 system. Neuroimage 46, 64–72 10.1016/j.neuroimage.2009.02.006 19233295

[B44] Kovelman I. Baker S. A. Petitto L. A. (2008a). Bilingual and monolingual brains compared using fMRI: is there a neurological signature of bilingualism? J. Cogn. Neurosci. 20, 1–17 10.1162/jocn.2008.20011 17919083 PMC2643466

[B45] Kovelman I. Baker S. A. Petitto L. A. (2008b). Age of first bilingual language exposure as a new window into bilingual reading development. Bilingualism Lang. Cognit. 11, 203–223 10.1017/S1366728908003386 19823598 PMC2759761

[B46] Kovelman I. Shalinsky M. H. Berens M. S. Petitto L.-A. (2008c). Shining new light on the brain's “Bilingual Signature:” a functional near infrared spectroscopy investigation of semantic processing. Neuroimage 39, 1457–1471 10.1016/j.neuroimage.2007.10.017 18054251 PMC2249758

[B48] Kovelman I. Shalinsky M. H. White K. S. Schmitt S. N. Berens M. S. Paymer N. Petitto L.-A. (2009). Dual language use in sign-speech bimodal bilinguals: fNIRS brain-imaging evidence. Brain Lang. 109, 112–123 10.1016/j.bandl.2008.09.008 18976807 PMC2749876

[B51] MacSweeney M. Woll B. Campbell R. Calvert G. A. McGuire P. K. David A. S. (2002a). Neural correlates of British sign language comprehension: spatial processing demands of topographic language. J. Cogn. Neurosci. 14, 1064–1075 10.1162/089892902320474517 12419129

[B52] MacSweeney M. Woll B. Campbell R. McGuire P. K. David A. S. Williams S. C. R. (2002b). Neural systems underlying British Sign Language and audio-visual English processing in native users. Brain 125(pt 7), 1583–1593 10.1093/brain/awf153 12077007

[B53] Mechelli A. Crinion J. T. Noppeney U. O'Doherty J. Ashburner J. Frackowiak R. S. (2004). Neurolinguistics: structural plasticity in the bilingual brain. Nature 431, 757–757 10.1038/431757a 15483594

[B55] Neville H. J. Coffey S. A. Lawson D. S. Fischer A. Emmorey K. Bellugi U. (1997). Neural systems mediating american sign language: effects of sensory experience and age of acquisition. Brain Lang. 57, 285–308 10.1006/brln.1997.1739 9126418

[B56] Newman A. J. Bavelier D. Corina D. Jezzard P. Neville H. J. (2002). A critical period for right hemisphere recruitment in American Sign Language processing. Nat. Neurosci. 5, 76–80 10.1038/nn775 11753419

[B57] Oldfield R. C. (1971). The assessment and analysis of handedness: the Edinburgh inventory. Neuropsychologia 9, 97–113 10.1016/0028-3932(71)90067-4 5146491

[B58] Paradis J. Nicoladis E. (2007). The influence of dominance and sociolinguistic context on bilingual preschoolers' language choice. Int. J. Biling. Educ. Biling. 10, 277–297 10.2167/beb444.0

[B60] Penhune V. B. Cismaru R. Dorsaint-Pierre R. Petitto L. A. Zatorre R. J. (2003). The morphometry of auditory cortex in the congenitally deaf measured using MRI. Neuroimage 20, 1215–1225 10.1016/S1053-8119(03)00373-2 14568490

[B62] Petitto L. A. (2005). How the Brain Begets Language. New York, NY: The Cambridge Companion to Chomsky 10.1017/CCOL0521780136.005

[B64] Petitto L. A. Holowka S. Sergio L. E. Ostry D. (2001b). Language rhythms in baby hand movements. Nature 413, 35–36 10.1038/35092613 11544514

[B63] Petitto L. A. Katerelos M. Levy B. G. Gauna K. Tétreault K. Ferraro V. (2001a). Bilingual signed and spoken language acquisition from birth: implications for the mechanisms underlying early bilingual language acquisition. J. Child Lang. 28, 453–496 10.1017/S0305000901004718 11449947

[B65] Petitto L. A. Kovelman I. (2003). The bilingual paradox: how signing-speaking bilingual children help us to resolve it and teach us about the brain's mechanisms underlying all language acquisition. Learn. Lang. 8, 5–19

[B66] Petitto L. A. Marentette P. F. (1991). Babbling in the manual mode: evidence for the ontogeny of language. Science 251, 1493–1496 10.1126/science.2006424 2006424

[B67] Petitto L. A. Zatorre R. J. Gauna K. Nikelski E. J. Dostie D. Evans A. C. (2000). Speech-like cerebral activity in profoundly deaf people processing signed languages: implications for the neural basis of human language. Proc. Natl. Acad. Sci. U.S.A. 97, 13961–13966 10.1073/pnas.97.25.13961 11106400 PMC17683

[B68] Poeppel D. Emmorey K. Hickok G. Pylkkänen L. (2012). Towards a new neurobiology of language. J. Neurosci. 32, 14125–14131 10.1523/JNEUROSCI.3244-12.2012 23055482 PMC3495005

[B70] Senghas R. J. Kegl J. (1994). Social considerations in the emergence of idioma de Signos Nicaragüense (Nicaraguan Sign Language). Signpost 7, 40–46

[B71] Shalinsky M. H. Kovelman I. Berens M. S. Petitto L. A. (2009). Exploring cognitive functions in babies children and adults with Near Infrared Spectroscopy. J. Vis. Exp. 10.3791/1268 19638948 PMC2780028

[B72] Schembri A. Johnston T. (2004). Sociolinguistic variation in Auslan (Australian Sign Language): A research project in progress. Deaf Worlds 20, S78–S90

[B73] Tak S. Ye J. C. (2014). Statistical analysis of fNIRS data: a comprehensive review. Neuroimage 85, 72–91 10.1016/j.neuroimage.2013.06.016 23774396

[B75] Wartenburger I. Heekeren H. R. Abutalebi J. Cappa S. F. Villringer A. Perani D. (2003). Early setting of grammatical processing in the bilingual brain. Neuron 37, 159–170 10.1016/S0896-6273(02)01150-9 12526781

[B76] Xiao Z. Zhang J. X. Wang X. Wu R. Hu X. Weng X. (2005). Differential activity in left inferior frontal gyrus for pseudowords and real words: an event-related fMRI study on auditory lexical decision. Hum. Brain Mapp. 25, 212–221 10.1002/hbm.20105 15846769 PMC6871751

[B77] Xue G. Poldrack R. A. (2007). The neural substrates of visual perceptual learning of words: implications for the visual word form area hypothesis. J. Cogn. Neurosci.19, 1643–1655 10.1162/jocn.2007.19.10.1643 18271738

[B78] Zatorre R. J. Meyer E. Gjedde A. Evans A. C. (1996). PET studies of phonetic processing of speech: review, replication, and reanalysis. Cereb. Cortex 6, 21–30 10.1093/cercor/6.1.21 8670635

